# Role of PGE_2_ in Asthma and Nonasthmatic Eosinophilic Bronchitis

**DOI:** 10.1155/2012/645383

**Published:** 2012-03-19

**Authors:** Beatriz Sastre, Victoria del Pozo

**Affiliations:** Immunology Department, IIS-Fundación Jiménez Díaz and CIBER of Respiratory Diseases, 28040 Madrid, Spain

## Abstract

Eosinophilic bronchitis is a common cause of chronic cough, which like asthma is characterized by sputum eosinophilia, but unlike asthma there is no variable airflow obstruction or airway hyperresponsiveness. Several studies suggest that prostaglandins may play an important role in orchestrating interactions between different cells in several inflammatory diseases such as asthma. PGE_2_ is important because of the multiplicity of its effects on immune response in respiratory diseases; however, respiratory system appears to be unique in that PGE_2_ has beneficial effects. We described that the difference in airway function observed in patients with eosinophilic bronchitis and asthma could be due to differences in PGE_2_ production. PGE_2_ present in induced sputum supernatant from NAEB patients decreases BSMC proliferation, probably due to simultaneous stimulation of EP2 and EP4 receptors with inhibitory activity. This protective effect of PGE_2_ may not only be the result of a direct action exerted on airway smooth-muscle proliferation but may also be attributable to the other anti-inflammatory actions.

## 1. Introduction

Asthma has consistently been reported as a major cause of chronic cough [1]. The development of noninvasive assessment of airway inflammation led to the identification of a condition that manifests chronic cough in individuals without the abnormalities of airway function that characterize asthma, but with sputum eosinophilia. This condition was named nonasthmatic eosinophilic bronchitis (NAEB) [2]. The reason for the absence of airway hyperresponsiveness in the NAEB remains unclear. Inflammation of the airways, with recruitment and activation of T lymphocytes, eosinophils, and mast cells and release of inflammatory mediators, plays an important role in the pathophysiology of asthma and NAEB. Among lipid mediators, PGE_2_ is a mediator thought to have an important role.

This paper shall summarize our current knowledge of the role of PGE_2_ in lung and in respiratory disease such as asthma and nonasthmatic eosinophilic bronchitis.

## 2. PGE_2_ Biosynthesis

Several studies suggest that prostaglandins may play an important role in orchestrating interactions between different cells in several inflammatory diseases such as asthma and rheumatoid arthritis.

Although the term prostaglandin was coined in the 1930s by Von Euler [3], and Bergstrom and Samuelsson defined the structure of two first prostaglandins in 1960 [4], the full structure of prostaglandins was not identified until 1965 by Orloff. Prostaglandins are arachidonic acid (AA) metabolites which result from enzymes with cyclooxygenase (COX) activity [5]. These metabolites are small lipidic molecules implicated in the regulation of many different processes in the organism. Their production begins with the liberation of AA from membrane phospholipids by phospholipase A2 in response to inflammatory stimuli [6]. AA is then transformed into prostaglandin H2 (PGH_2_) by COXs, which is the first step in eicosanoid biosynthesis. PGH_2_ is an unstable molecule that is transformed into several biologically active prostaglandins through specific enzymes with different tissue and cellular expression pattern [7].

Two isoforms of COX have been identified, and they are classified as COX-1 and COX-2. The main differences between them are their expression regulation and tissue distribution. In terms of expression, COX-1 is constitutively expressed in cells in which prostaglandins exert physiological functions, while COX-2 expression is enhanced after inflammatory stimuli [8], such as LPS, several proinflammatory cytokines (tumor necrosis factor-alpha, interleukin-1-beta), growth factors, or tumoral promoter as PMA [4, 9]. Both isoforms catalyze similar reactions although they are codified by different genes [10]. COX-1 is associated with immediate biosynthesis of prostaglandins (several minutes after stimulation) which develop homeostatic functions; COX-2 is linked to delayed biosynthesis of prostaglandins (several hours after stimulus) which exert pathological effects. Other difference is the cellular localization; thus, COX-1 is expressed in endoplasmic reticulo, whereas COX-2 is situated in perinuclear membrane [7, 11].

PGE_2_ is the most abundantly produced prostanoid in the body and has been shown to play an important role in regulating inflammatory processes. PGE_2_ production is largely dependent upon three enzymatic reactions: generation of arachidonic acid from membrane glycerophospholipids via phospholipase A2, conversion of AA to an unstable intermediate prostanoid (PGH_2_) by COXs, and metabolism of prostaglandin H_2_ to prostaglandin E_2_ via prostaglandin E synthase [12].

There are three enzymes that catalyze PGE_2_ generation starting from PGH_2_, namely, membrane-bound PGES (mPGES)-1 [13], mPGES-2 [14], and cytosolic PGE (cPGES) [15] which constitute two biosynthetic pathways to PGE_2_ secretion; on one hand, COX-1/cPGES, the pathway implicated in homeostasis and does not play an important role in PGE_2_ production [16], and on the other, COX-2/mPGES-1, the pathway associated with delayed response, inflammation, and pathology [17], and that is essential for basal production in some organs as brain, spleen, and stomach (at least in mice) [18].

Major cellular sources of PGE_2_ include epithelial cells, endothelial cells, airway smooth muscle, and monocytes/macrophages [19].

## 3. PGE_2_ Receptors

PGE_2_ exerts its effects by acting on a group of rhodopsin-type G-protein-coupled membrane receptors (GPCRs) termed E-prostanoid (EP) receptors. There are four GPCR subtypes: EP1, EP2, EP3, and EP4 [20, 21]. Expression regulation of the various subtypes of EP receptor by several agents, such as inflammatory stimuli, or even PGE_2_ itself, enables PGE_2_ to affect tissues in a very specific and diverse manner [22, 23]. These subtypes of EP receptor differ in the intracellular signaling [24]. The expression or a combination of receptors, each which may be differentially expressed in a number of tissues (i.e., airway smooth muscle, neurons, or immune effector cells), results in a specific physiological response. These receptors could be classified according to their intracellular signaling and second messenger. EP1 receptor activation leads to an increase in intracellular calcium, usually coupled to Gq protein, and its stimulation is linked to phospholipase C [25]. EP2 and EP4 receptors couple to Gs, and activation of these receptors results in stimulation of adenylyl cyclase and increases intracellular cAMP [26]. The major signaling pathway described for the EP3 receptor is mediated by G*α*i and leads to a reduction in intracellular cAMP levels. However, several EP3 receptor isoforms generated by alternative splicing from the single EP3 receptor gene have been identified, and the intracellular signal may differ [27]. Some of these isoforms of the EP3 receptor coupled to multiple G proteins produced either inhibition of adenylate cyclase and calcium mobilization or stimulation of adenylate cyclase activity [26]. Thus, accumulation of cAMP promoted by EP2 and EP4 receptors is associated with the inhibition of effector cell functions; however, EP1 and some isoforms of the EP3 receptor that increase intracellular calcium could be linked to promotion of cellular activation [20].

In pathological conditions, the role of these receptors is determined by pattern expression, ligand affinity, and their differential coupling to transduction signaling pathways in which the cellular context of the receptor is very relevant.

## 4. PGE_2_ Effects on Respiratory System

PGE_2_ is almost ubiquitous in humans and evokes potent diverse actions. It regulates several functions in the major human systems, including the gastrointestinal, reproductive, neuroendocrine, and immune systems [28].

It is in the area of inflammation that the actions of PGE_2_ are most diverse because of the many specialized cell types as well as the complex and sometimes seemingly opposing actions that make that PGE_2_ one of the most heterogeneous eicosanoids. This complexity reflects differences between endogenous formation and action *versus* pharmacological and exogenous addition of PGE_2_ *in vivo* and *in vitro* [29].

PGE_2_ acts like a pleiotropic prostaglandin with stimulating or inhibiting properties and could be classified as a regulatory element of immunity [30]. An example of this stimulating/inhibiting polarity is the opposite actions that PGE_2_ exerts in the cardiovascular and immune system, where PGE_2_ is able to enhance the inflammation caused by leukotrienes as well as inhibit the release of mediators and regulate monocyte macrophages and dendritic cells [31].

Although PGE_2_ acts in multiple systems, we will focus our attention on the role that this prostaglandin plays in the inflammatory process linked to respiratory system.

PGE_2_ is the major metabolite in the lower respiratory tract. In this system, epithelium and airway smooth muscle are the principal source of PGE_2_ [32], though fibroblast, alveolar macrophages, and pulmonary endothelial cells also produce it [33, 34]. PGE_2_ is important because of the multiplicity of its effects on immune response in respiratory diseases.

PGE_2_ is commonly presumed to be a proinflammatory mediator and has been implicated in several inflammatory disease conditions, including rheumatoid arthritis [35]; however, PGE_2_ has protective effects in different organs, and respiratory system appears to be one of them in that PGE_2_ has beneficial effects [26, 36–38].

During the 1970s, PGE_2_ was shown to protect against bronchoconstriction produced by ultrasonically nebulized distilled water [39] and exercise-induced asthma [40]. In the early 1990s, Pavord et al. [41] showed that inhaled PGE_2_ protected against bronchial hyperreactivity to sodium metabisulphite in which bronchoconstriction is thought to be neurally mediated. In this study, furosemide protected against bronchoconstrictor challenges in asthma, and this effect may be mediated through PGE_2_. Multiple subsequent studies have observed the bronchodilator effect of PGE_2_ in normal subjects [42] and patients with asthma and chronic bronchitis [43], showing that PGE_2_ attenuates bronchoconstriction, possibly inhibiting the release of the bronchoconstrictor mediators which are responsible for exercise bronchoconstriction. This prostanoid inhibits early and late allergen-induced bronchoconstriction, increasing the relaxation of airway smooth muscle and inhibiting the release of mast-cell mediators and the recruitment of inflammatory cells [34]. Moreover, PGE_2_ also decreases or inhibits the accompanying bronchial hyperresponsiveness to methacholine [36].

All these positive effects of PGE_2_ are mainly mediated through EP2 and EP4 receptors [44]. PGE_2_ can mediate bronchodilation via the EP2 receptor [45] and also anti-inflammatory effects via the EP2 and/or the EP4 receptor [46]. Also, EP2 plays an important role in aspirin-intolerant asthma because a reduction in released PGE_2_ and lower expression of its EP2 receptor provoked an increase in inflammatory process in the airways of these patients [47]. However, PGE_2_ also induces irritation of the upper airway, resulting in a reflex cough and enhancing the response to capsaicin. The coughing induced by this prostanoid is caused mainly, if not solely, by activation of the EP3 receptor, and in bronchoconstrictor effects are implicated by both EP1 and EP3 receptors [48]. 

## 5. PGE_2_ and Inflammatory Cells

Immune cells produce a variety of prostaglandins that have both proinflammatory and anti-inflammatory effects [49]. Eosinophils are one of predominant inflammatory cells in the lungs of asthmatic patients, and changes in the number and degree of lung eosinophils probably influence disease severity. Peacock and colleagues demonstrated that PGE_2_ suppresses eosinophil apoptosis, and this is likely mediated by interaction with the EP2 receptor subtype in eosinophils [50], by contrast, misoprostol (a PGE_2_ analogue) inhibits eosinophil survival *in vitro* [51]. Nevertheless, in recent years, all studies have shown a negative regulator role of PGE_2_ on eosinophils. This prostanoid directly controls eosinophils' migration on the cellular levels and is highly potent and efficacious. This effect is brought about mainly by EP2 receptor involving PI3K- and PKC-dependent pathways [52]. Furthermore, PGE_2_ not only attenuates eosinophil trafficking but also abolishes the production of reactive oxygen species, Ca^2+^ responses, and upregulation of adhesion molecules through the EP4 receptor [53]. EP4 does not seem to depend on activation of the adenylyl cyclase/PKA pathway. Its stimulation causes phosphorylation of extracellular signal-regulated kinases (ERKs) through a PI3K-dependent mechanism [53]. So, an alternative EP2/EP4 signaling pathway in which both PI3K and PKC activation are implicated has been postulated.

PGE_2_ also acts on T cells and alveolar macrophages. It is able to decrease proliferation of lymphocytes, subsequently decreasing the production of Th2 cytokines [54]. In another study, PGE_2_ produced an increase of IL-10 expression, an important regulatory cytokine [55]. In contrast, PGE_2_ positively controls the regulatory T cells (Treg) which are pivotal in suppressing immune responses and maintaining tolerance. PGE_2_ upregulates FOXP3 mRNA and protein expression and enhances FOXP3 promoter activity [56]. PGE_2_ also enhances Ig class switch to IgE in B cells [57].

PGE_2_ is a route through which airway epithelial cells (AECs) modulate specific cellular subtypes such as dendritic cells. Along these lines, Schmidt and colleagues [58] demonstrated that epithelial cells, through the constitutive secretion of PGE_2_, drive DCs to adopt an anti-inflammatory phenotype in an EP4 receptor-dependent manner coupled to cAMP production. As a result, PGE_2_-EP4 receptor signaling generates DCs with reduced proinflammatory properties (decreased production of TNF-*α* and enhancing IL-10 secretion), thereby limiting DC activation [58].

Also, in alveolar macrophages, it produces an increase of IL-10 production and a decrease of TNF-*α* levels generated by alveolar macrophages [59, 60].

## 6. PGE_2_ and Lung Structure

As mentioned above, the principal sources of PGE_2_ in airways are the epithelial, endothelial, fibroblast, and smooth muscle cells. The epithelium is the first barrier to protect against injury. Its cells create an anti-inflammatory microenvironment that modulates the phenotype of local antigen-presenting cells (APCs), regulating the activation of local professional immune cells. A chronic state of inflammation and wound healing exists in the lung as a part of asthma pathology. Moreover, fibroblasts actively migrate and proliferate, synthesize and secrete extracellular matrix components, and differentiate into myofibroblasts. In this process, PGE_2_ exerts significant negative regulatory functions by inhibiting fibroblast migration and chemotaxis with a dominant role of EP2 receptors in the cAMP-dependent inhibitory effects [61, 62], thereby decreasing fibroblast proliferation through Epac-1 and subsequent Rap1 activation (cAMP effectors) in a manner which is independent of ERK1/2 participation [63]; furthermore, PGE_2_ inhibits collagen expression by a PKA-mediated process which is linked to EP2 and EP4 receptors [63]. Impaired *ex vivo* COX-2 function and PGE_2_ synthesis that characterize fibroblast during the evolution of airway fibrosis may likewise promote fibrogenesis [64]. However, this inhibition of fibrosis may also occur through an inhibition of fibroblast PAR1 expression, by PAR2-mediated generation of PGE_2_, one of the protease-activated receptors coupled to G protein that possesses a unique mechanism of activation and induces COX activation in a variety of cell types [65]. PAR-1 signalling is a well-known profibrotic pathway [66], so inhibition of PAR1 expression diminished pulmonary fibrosis.

PGE_2_ has complex effects on airway tone, and the existence of several E-prostanoid receptors, each one with different signalling characteristics, has provided a possible explanation for the seemingly contradictory actions of this lipid mediator. Potent relaxant effects on airway smooth muscle have been observed; however, human studies with aerosolized PGE_2_ have demonstrated inconsistent effects on airway tone, with most asthmatics showing a bronchodilator response but some developing profound bronchoconstriction requiring beta agonist rescue [36, 41, 43, 48].

## 7. Asthma and Nonasthmatic Eosinophilic Bronchitis

Asthma is a complex chronic disease of the airways that has been estimated to affect over 300 million people worldwide, and the burden is likely to rise in the coming decades [64, 67].

The inflammatory process in asthma is characterized by inflammatory cells, mainly eosinophils, mast cells, basophils, macrophages, and Th2 cells. These cells are involved in the development of another hallmark of asthma as airway hyperresponsiveness (AHR), reversible airway obstruction, cough, mucus secretion, and structural changes by releasing inflammatory mediators such as cytokines, chemokines, growth factors, and chemical and lipid mediators [68, 69]. The complex pathogenesis of asthma is contributed to by various cellular responses, based on the dysregulated interaction between innate and adaptative immune systems.

Chronic cough is a common reason for referral to a specialist, and asthma has consistently been reported among the most common causes of chronic cough, accounting for about 25% of such cases in adult nonsmokers [1, 70, 71]. The development of sputum induction has provided a safe noninvasive method of assessing airway inflammation. One of the most interesting early observations made using this method was the identification of a group of patients with sputum eosinophilia identical to that seen asthma, but with normal airway function. The physiological features of this condition were different from those of asthma, and Gibson and colleagues suggested that the new disease should be known as eosinophilic bronchitis [2]. The cough in patients with nonasthmatic eosinophilic bronchitis (NAEB) responds well to inhaled corticosteroids, though the same is not the case for cough in patients without sputum eosinophilia. NAEB is responsible for about 10% of cases of isolated chronic cough referred for specialist investigation [72].

The etiology of asthma and NAEB is usually unknown, although both can be associated with exposure to an occupational sensitizer or to common inhaled allergens [73–75]; thus, the triggers that cause eosinophilic bronchitis without asthma are similar to the triggers of eosinophilic bronchitis with asthma.

In patients with eosinophilic bronchitis, there is a clear dissociation between sputum eosinophilia and airway hyperresponsiveness. The pathophysiology of eosinophilic bronchitis and the reason for the absence of airway hyperresponsiveness in this disease remains unclear. One possible explanation is that the eosinophilic airway inflammation is less active than in asthma, with less release of effector mediators. Several studies using different techniques to assess airway inflammation have shown that the inflammatory component is very similar in patients with asthma and NAEB [76]. Indeed, asthma and eosinophilic bronchitis share many immunopathologic features including increased number of eosinophils and mast cells in the superficial airway. In addition to airway eosinophilia, both conditions are associated with reticular basement membrane thickening and similar number of subepithelial T lymphocytes, mast cells, and macrophages [76]. Eosinophilic bronchitis is a disease characterized by increased expression of Th2 cytokines (IL-4, IL-5, IL-10, and IL-13) [77, 78]. These data point to a dissociation between T-cell activation and the abnormalities in airway physiology that characterize asthma [79]. Such results suggest that Th2-mediated cytokines are closely linked to eosinophilic inflammation, though they are not necessarily associated with the physiologic hallmarks of the asthmatic phenotype.

One aspect of the inflammatory response that might be particularly important is the localization of mast cells in the airway wall, since they are present within the airway smooth muscle in asthma but not in NAEB [80]. Moreover, in subjects with eosinophilic bronchitis, CXCL8 and CXCL10 concentrations were elevated in airway secretions. These chemokines may play a key role in mast cell recruitment to the superficial airway in this condition [81]. Siddiqui and colleagues have demonstrated that mast cells are microlocalized within the airway smooth muscle bundle in asthma, which is associated with airway hyperresponsiveness [82]. Mast cells produce a variety of mediators that may interact with bronchial smooth muscle and subsequently become hyperresponsive to constrictive stimuli and proliferation [83].

## 8. PGE_2_ in Asthma and NAEB

We recently found that induced sputum prostaglandin E2 (PGE_2_) concentrations are strikingly increased in subjects with NAEB as compared with asthmatic and healthy subjects [78]. This study illustrates that, like asthma, there is active airway inflammation in eosinophilic bronchitis with release of different inflammatory mediators. These data suggest that the differences in airway function observed in subjects with NAEB and asthma may be due to differences in PGE_2_ production.

Brightling et al. [84] also found numerically higher levels of PGE_2_ in sputum from patients with eosinophilic bronchitis, though the differences were not statistically significant as compared with asthma patients. Recently, elevated sputum PGE_2_ concentrations have been found in all patients with chronic cough [73], although in this study cough variant asthma and eosinophilic bronchitis patients were included in the same group.

The differences in sputum PGE_2_ concentration between asthmatic and eosinophilic bronchitis patients may be the result of the implication of a different degradation kinetic or activity of enzymes in the synthesis and/or degradation of products of arachidonic acid metabolism. It has been recently shown that Th2 cytokines have specific effects on PGE synthase 1 and 15-PGDH enzymes in airway human epithelial cells decreasing PGE_2_ [85].

We postulate that PGE_2_ elevation in patients with eosinophilic bronchitis may have protective effects against the development of bronchial hyperresponsiveness. The fact that PGE_2_ and its analogue have a number of bronchoprotective and anti-inflammatory effects *in vitro *[37] or after inhalation [36], as well as on allergen-induced airway responses and airway inflammation in atopic asthma [38, 86], would support such a protective role. An imbalance in the ratio of bronchoconstrictor (LTC_4_) and bronchoprotective (PGE_2_) lipid mediators may have a role in the pathogenesis of eosinophilic bronchitis.

PGE_2_ can perform contrasting activities, which can thus lead to bronchodilation and act as anti-inflammatory or proinflammatory mediator substances in the lung [26]. Recently, several studies showed that the PGE_2_ protective actions were mediated in large part by the EP_2_ receptors [87, 88]. This protective action of PGE_2_ might not only result in a direct effect on airway smooth-muscle relaxation, but also in the inhibition of many inflammatory processes [89]. These data may explain why patients with eosinophilia have normal tests of variable airway obstruction and airway responsiveness and experience chronic cough due to high PGE_2_ levels [90].

The pathogenesis of eosinophilic bronchitis might be the opposite of that observed in aspirin-induced asthma. There is increasing evidence that, by inhibiting cyclooxygenase-1, the protective effect exerted by endogenous prostaglandin E_2_ on leukotriene generation by mast cells, eosinophils, and macrophages in the airways is removed in aspirin-induced asthma. In these patients with aspirin-induced bronchoconstriction, a low production of PGE_2_ has been observed, seemingly due to deficient COX-2 regulation and increased expression of leukotriene-C4 synthase [91].

## 9. Role of PGE_2_ in NAEB

Muscle hypertrophy and hyperplasia are characteristics of asthmatic airways, and this increased layer of bronchial smooth muscle contributes to AHR in asthmatic patients [92, 93]. We hypothesized that high PGE_2_ levels in the lower airways of NAEB patients constitute the essential regulatory mediator causing inhibition of bronchial smooth muscle cell proliferation (BSMC) and subsequent hyperresponsiveness [94]. Thus, we demonstrated that when BSMCs are cultured with induced sputum supernatant from NAEB patients, a strong inhibition of muscle cell proliferation takes place [94]. This inhibition was not due to the apoptotic effect of sputum supernatants or to differences in cytokine levels found in sputum. Formal proof of this finding was the addition of EP2 and EP4 antagonists to the culture recovery of the proliferation of smooth muscle cells. The lesser inhibition obtained from sputum of asthmatic patients may be explained by the absence or defectiveness of PGE_2_ production, as well as by differential EP2 and EP4 receptor expression from different pathologies. Similarly, Lundequist and colleagues recently described a role for PGE_2_ in protecting the pulmonary vasculature from remodeling dependent on more than one EP receptor [95].

**Figure 1:**
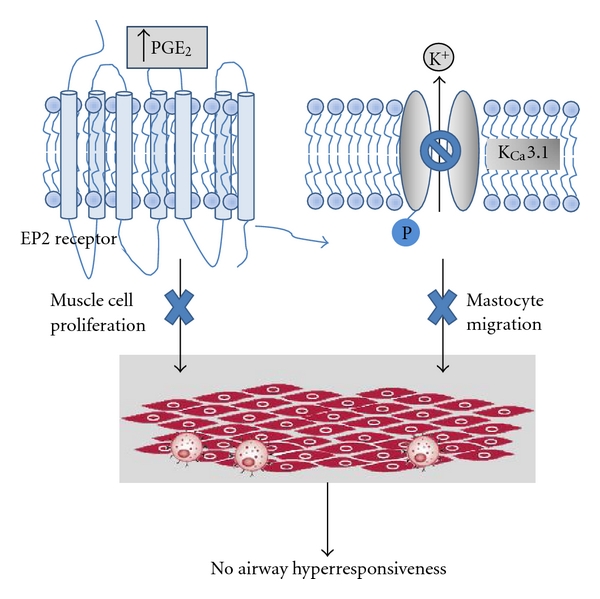
Hypothetical mechanisms through which PGE_2_ reduces the AHR and in NAEB. (a) The PGE_2_ decreases the smooth muscle proliferation producing a reduction of muscular hyperplasia, via EP2 and EP4 receptors; (b) The PGE_2_ closes the K_Ca_ 3.1 channel, preventing the migration of mastocytes by means of EP2. Both mechanisms will decrease or inhibit airway hyperresponsiveness, a relevant hallmark of asthma.

An alternative explanation of the differences in airway function between asthma and NAEB is the different localization of mast cells within the airway wall. Siddiqui and colleagues have demonstrated that mast cells are microlocalized within the airway smooth muscle bundle in asthma, and this is associated with AHR [82]. Mast cells produce a variety of mediators that may interact with BSM and subsequently become hyperresponsive to constrictive stimuli and proliferation [83]. We hypothesize that both mechanisms may act synergistically (Figure 1). Recently, Duffy and colleagues reported that the engagement of the EP2 receptor closes the K^+^ channel K_Ca_ 3.1 in human lung mast cells and attenuates their migration [96]; thus, PGE_2_ present in sputum supernatant from NAEB patients could close the K_Ca_ 3.1 channel and inhibit mast cell migration to the airway wall and subsequently bring about microlocalization within BSMC. Furthermore, in the inhibition of BSM proliferation produced by PGE_2_, the K^+^ channel K_Ca_ 3.1 may be implicated since activated K^+^ channels regulate human airway smooth muscle proliferation [97].

Airway smooth muscle cells are the major effector cells regulating bronchomotor tone in response to several mediators [98]. Some authors have reported that increased vascularity, reticular basement membrane thickening, and increased airway smooth muscle mass are features of both diseases [99, 100]. However, the same authors have recently reported that patients with asthma had airway wall thickening, as opposed to subjects with NAEB, who maintained airway patency without wall thickening [101]. In addition, AHR and altered airway geometry were found to be correlated in asthma patients. Maintained proximal airway patency in NAEB compared to the subjects with asthma may protect against the development of AHR. In line with this, Park et al. have reported that proximal airway wall thickening is not a feature of NAEB [102].

In conclusion, PGE_2_ present in induced sputum supernatant from NAEB patients decreases BSMC proliferation, probably due to simultaneous stimulation of EP2 and EP4 receptors with inhibitory activity. This protective effect of PGE_2_ may not only be the result of a direct action exerted on airway smooth-muscle proliferation but also may be attributable to the other anti-inflammatory actions. Thus, PGE_2_ agonist receptors may become a novel therapeutic approach for inflammatory respiratory diseases.
